# Collagen-Based Scaffolds for Volumetric Muscle Loss Regeneration

**DOI:** 10.3390/polym16233429

**Published:** 2024-12-06

**Authors:** Anna L. Luss, Maria M. Bobrova, Pavel P. Kulikov, Anton A. Keskinov

**Affiliations:** Federal State Budgetary Institution «Centre for Strategic Planning and Management of Biomedical Health Risks» of the Federal Medical and Biological Agency, Pogodinskaya st., b.10/1, 119121 Moscow, Russia; mbobrova@cspfmba.ru (M.M.B.); pkulikov@cspfmba.ru (P.P.K.); keskinov@cspfmba.ru (A.A.K.)

**Keywords:** collagen, hydrogel, scaffold, regeneration, tissue engineering

## Abstract

Volumetric muscle loss (VML) is a serious problem in healthcare that requires innovative solutions. Collagen and its derivatives are promising biomaterials for muscle tissue replacement due to their high biocompatibility, biodegradability, and lack of toxicity. This review comprehensively discusses collagen from various sources, its structural characteristics, cross-linking methods to obtain hydrogels, and approaches to incorporating various therapeutic molecules to create a biocomposite system with controlled release. Collagen-based scaffolds are promising constructs in tissue engineering and regenerative medicine. They can both perform their function independently and act as a depot for various biologically active substances (drugs, growth factors, genetic material, etc.). Collagen-based scaffolds for muscle volume restoration are three-dimensional constructs that support cell adhesion and proliferation and provide controlled release of therapeutic molecules. Various mechanical and biological properties of scaffolds can be achieved by cross-linking agents and bioactive molecules incorporated into the structure. This review highlights recent studies on collagen-based hydrogels for restoration of volumetric muscle loss.

## 1. Introduction

Skeletal muscles make up 40–45% of an adult’s body weight. Skeletal muscle injuries are common and can be caused not only by trauma and surgical interventions but also by genetic diseases such as Duchenne muscular dystrophy and amyotrophic lateral sclerosis [1,2].

Skeletal muscle tissue has a high potential for regeneration after injury due to muscle tissue stem cells—myosatellite cells [3]. They are activated in response to external stimuli, which can be either ordinary physical exercise or various injuries or damages. With minor injuries, they can either fuse with existing myofibrils or with each other, resulting in the growth and maturation of muscle fibers and promoting regeneration of the injured muscles [4,5].

Despite the presence of small wound regeneration mechanisms, volumetric muscle loss (VML), which exceeds 20% of the total skeletal tissue volume, leads to permanent functional limitations [6,7]. Since VML cannot be restored naturally, surgery is required. This can significantly affect the quality of patients’ lives, severely limiting the functionality of the musculoskeletal system.

Currently, the standard approach to VML regeneration involves transplantation of the missing area with healthy autologous tissue. Healthy muscle tissue from an uninjured area (usually the latissimus dorsi and gracilis) is transplanted to the area of muscle loss. Although these surgeries restore function in most cases, they can cause loss of innervation at the donor site. Additionally, up to 10% of these surgeries result in complications such as infection and necrosis [8]. The procedure is also associated with many problems, such as a lack of donor tissue, painful procedures, and loss of muscle donor site functionality [9].

Another clinically effective method is physical therapy. Physical therapy can indeed improve muscle recovery by strengthening intact muscle tissue, modulating the immune response, releasing growth factors, stimulating vascularization, and reducing scar formation. However, physical therapy does not provide significant muscle regeneration in VML [8]. Extracellular matrices are also promising for musculotendinous tissue repair. Currently, a clinical trial (NCT01292876) is underway to test extracellular matrix for skeletal muscle tissue repair. The study evaluated healing and return of function after implantation of an extracellular matrix device in 17 men and women enrolled in the University of Pittsburgh Department of Plastic and Reconstructive Surgery who suffered from skeletal muscle tissue loss injury [10]. One of the promising methods for inducing regeneration is the influence of a magnetic field. A time-dependent magnetic field improved the proliferation and differentiation of stem cells [11].

Tissue engineering based on natural, synthetic, and composite biomaterials provides a reliable platform for the development of scaffolds that promote skeletal muscle regeneration, restoration of muscle contractile strength, vascularization, and innervation [12].

Hydrogel-based scaffolds have unique properties, including swelling by absorbing water or biological fluids [13]. They can be adapted to the shape of the defect, thereby facilitating more efficient delivery of biologically active substances to the wound bed [14].

Hydrogels are three-dimensional networks composed of hydrophilic polymers cross-linked either through covalent bonds or held together through non-covalent physical interactions. The high hydrophilicity of hydrogels is due to the presence of hydrophilic fragments, such as carboxyl, amide, amino, and hydroxyl groups, distributed along the main chain of the polymer. In the swollen state, hydrogels are soft and rubber-like, having physical and mechanical characteristics similar to native tissue. In addition, many hydrogels, for example those based on polysaccharides, have a high level of biocompatibility [13].

Collagen is the most abundant protein in mammals, accounting for 25–45% of all proteins. The global hydrolyzed collagen market reached nearly USD 4 billion in 2023. The rapid growth of the market is due to its increasing usage in cosmetics as a moisturizing agent and in dietary supplements to combat the symptoms of osteoarthritis and intestinal diseases. The native properties of collagen, including biodegradation to form non-toxic compounds, make it most suitable for the development of biomedical constructions to promote cell adhesion and proliferation. This advantage makes it suitable for tissue engineering and regenerative medicine. The global market for collagen materials for tissue engineering is expected to be worth more than USD 6 billion by 2032 [15].

**Figure 1:**
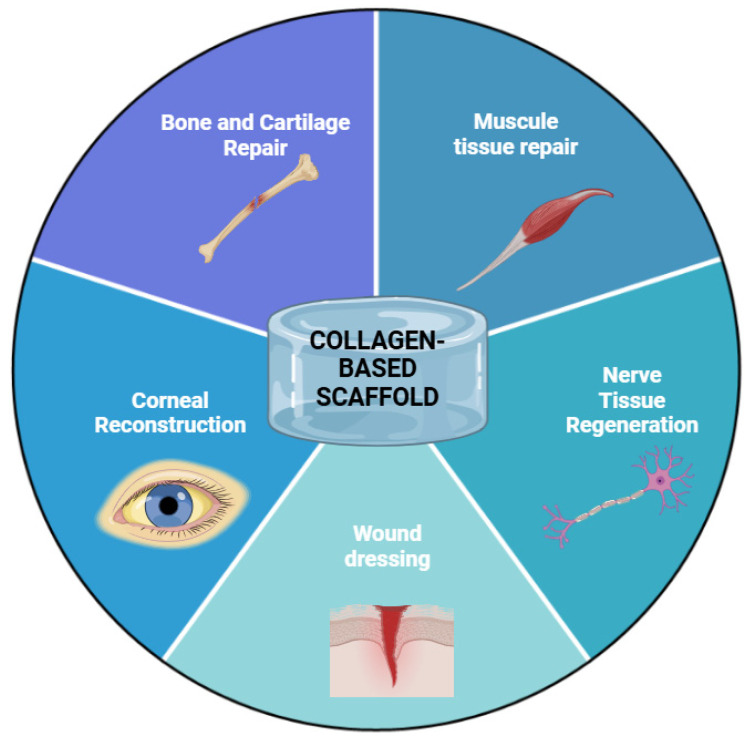
Application of collagen-based scaffolds.

Collagen-based scaffolds can be used in various fields of medicine, such as to restore volumetric muscle losses [16], as a basis for bone and joint implants [17,18], in the treatment of corneal diseases [19,20,21], for the restoration of nerve tissue [22,23], and as wound dressings [24,25] (Figure 1).

Today, there are quite a lot of commercially available collagen-based medical products for regenerative medicine. The main areas are bone tissue regeneration in dentistry and hemostatic sponges. As a rule, hemostatic sponges are composed of purified native human or porcine collagen, while composites for bone and soft tissue replacement contain additional components, such as proteins, glycosaminoglycans, antiseptics, etc. (Table 1).

However, natural collagen also has several limitations, such as the lack of standardization of the resulting raw materials in terms of purity and quality, and a high risk of contamination with various pathogens. Additionally, there is significant variability in the structure and mechanical properties of hydrogels derived from animal type I collagen, depending on the tissue source and the age of the animal, which influences cell morphology and proliferation [26].

## 2. Structure and Sources of Collagen

**Figure 2:**
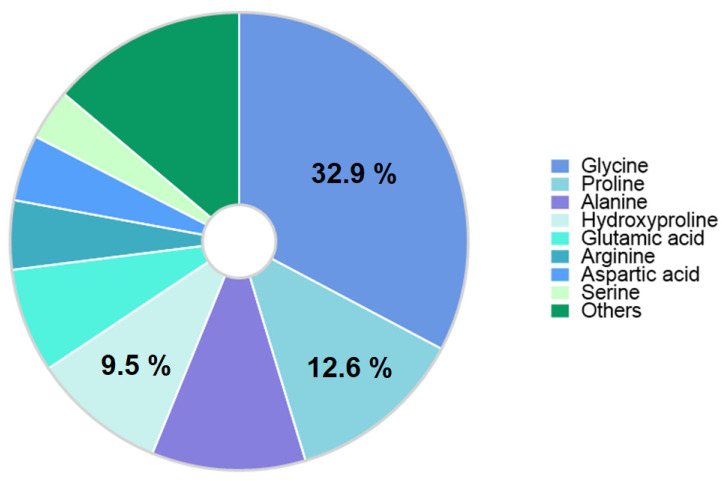
Amino acid content of human collagen.

Collagen is the most important extracellular matrix protein synthesized in the human body, the primary structure of which is formed as an α-chain. The main amino acids in the chain are glycine (~33%) and proline and hydroxyproline (~22%) (Figure 2). Each α-chain consists of approximately 1014 amino acids with a molecular weight of approximately 100 kDa.

**Figure 3:**
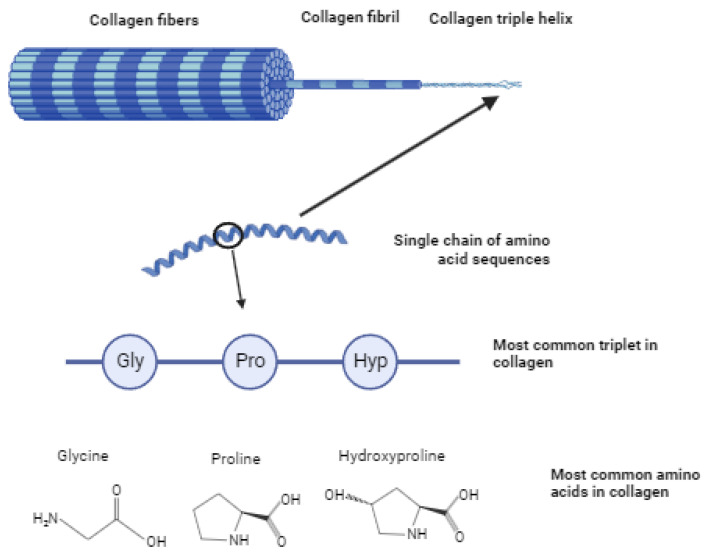
Structure of collagen fibers.

These chains are twisted into a left-handed helix with three amino acids per turn (secondary structure). The chains are twisted around each other in a triple helix, forming a rigid structure (tertiary structure). The collagen triple helix consists of a repeating sequence of amino acids (Gly-X-Y)_n_. Theoretically, there are more than 400 possible Gly-X-Y triplets, but analysis of fibrillar and non-fibrillar collagens shows that only certain amino acid sequences are found within the protein. The most common triplet is Gly-Pro-Hyp, which also provides maximum triple helix stability [27]. Collagen also contains the Arg-Gly-Asp sequence, or RGD-motif, which ensures cell adhesion to the extracellular matrix. Cell adhesion proteins of the integrin family recognize and bind to this sequence and activate processes such as cell attachment, proliferation, and differentiation [28,29]. The supercoil (quaternary structure) is the basic structure of collagen (Figure 3). This collagen structure is very stable due to intramolecular hydrogen bonds between glycines in adjacent chains [30].

Collagen molecules are classified into 28 different types, which are grouped into eight families based on their structure, bonding, and predominant distribution in the human body. Classifications include fibril-forming, basement membrane, microfibrillar, anchoring fibrils, hexagonal mesh-forming, fibril-associated collagens with interrupted triple helix [FACIT], transmembrane, and multiplexins [31]. Differences in collagen types are determined by the following parameters: the way structural elements are connected, the presence of additional domains, the way collagen fibers are assembled, and function in the body. More than 90% of all collagen in higher organisms is collagen types I, II, III, and IV [32].

Collagen is obtained from animal and plant sources. The most common animal sources are bovine, porcine, and human collagen [33]. Among these animal sources, bovine collagen is commonly used as a temporary wound dressing as well as for burn treatment. It is widely used due to its high biocompatibility. Porcine collagen matrices can be utilized for soft tissue transplantation. Constructions based on porcine and bovine pericardium are used in cardiovascular surgery as prosthetic heart valves [34]. In this case, decellularized pericardium is most often applied, which does not contain cellular material, and the collagen in its composition retains the native structure [35,36,37]. However, porcine and bovine collagen can cause an immune response or zoonotic infection (e.g., foot-and-mouth disease, mad cow disease, etc.). It is also worth considering the religious aspect, as Hinduism, Islam, Judaism, and other religions limit the use of porcine and bovine collagen [38].

In the last decade, collagen from marine animals has attracted widespread interest [39,40,41,42]. Type I fish collagen is unique in its extremely high solubility in dilute acid compared to mammalian collagen [43]. Duck’s feet-derived collagen is biocompatible, stable, and free from zoonotic infection, and contains 97% type I collagen [38].

New methods of collagen synthesis also seem interesting. For example, the synthesis of recombinant collagen using bacteria. Hydrophilic repeats (Gly-X-Y)_n_ are expressed in *Pichia pastoris* bacteria. After purification using ultrafiltration and ion exchange chromatography, high-purity collagen was obtained. Analysis of molecular weight and amino acid composition, as well as a collagenase degradation test, confirmed the similarity of the resulting collagen with human type I collagen. The synthesized collagen has a network structure and is highly cytocompatible [44,45]. In addition, recombinant collagen synthesis has been successfully demonstrated in both prokaryotic and eukaryotic cells, including *Escherichia coli*, fungi, plants, and animal systems [46].

However, the utilization of pure collagen to replace VML is not possible without additional modifications due to its biological instability and rapid destruction by enzymes.

## 3. Hydrogel Fundamental Properties for Tissue Regeneration

**Figure 4:**
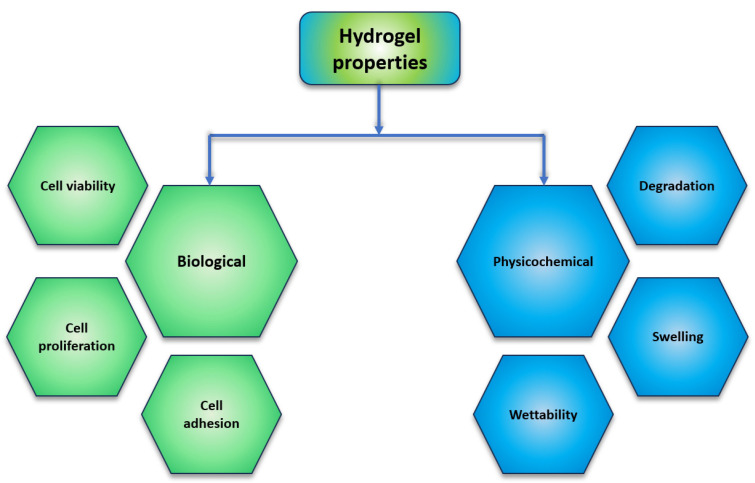
Hydrogel fundamental properties for tissue regeneration.

New tissue growth requires appropriate hydrogel design parameters. Hydrogels must be biocompatible to function properly in vivo, to avoid cell damage and immune system reactions, and biodegradable to replace new tissue. Like the natural extracellular matrix, hydrogels should facilitate cell migration, proliferation, and adhesion. Mechanical properties will depend on the swelling degree, degradation rate, and cross-linking degree of the hydrogel. All hydrogel properties can be roughly divided into biological and physicochemical properties (Figure 4).

### 3.1. Cell Adhesion and Migration

Cell migration is a fundamental phenomenon in wound healing and tissue regeneration. Hydrogels play the role of the extracellular matrix [47]. In nature, migration through the extracellular matrix occurs predominantly in directions determined by the internal architecture of the matrix. Pathways that are either inherently present in tissues (e.g., between collagen bundles, or along neural circuits or small blood vessels) or develop during extracellular matrix remodeling provide rapid cell migration [48]. Cell adhesion and migration are critical processes in tissue engineering and regenerative medicine. These processes are necessary for the creation of functional and well-integrated tissue substitutes, since cell adhesion refers to the initial interaction between host site cells and the hydrogel surface [49]. Hydrogels have various functional groups and can be loaded with growth factors that stimulate signaling molecules involved in the process of dynamic cell adhesion. Cell adhesion can be improved by surface modification, bio-functionalization, and surface roughness [50,51].

### 3.2. Cell Proliferation

Hydrogels have multiple accessible functional groups and active sites that promote proper cell attachment and lead to cell proliferation, differentiation, and migration to repair and regenerate damaged tissues or wounds. Hydrogels provide a sufficient microenvironment that supports the development of new tissue through cell differentiation and proliferation [52]. The microenvironment created by hydrogels regulates cell proliferation and differentiation, and it can be modified by incorporating growth factors, hydrogel structure, etc. The microenvironment can direct cell division, differentiation, and ultimately the production of functional tissues that are very similar to their native tissue [53].

### 3.3. Cell Viability

Cell viability is critical for cell proliferation or differentiation for the regeneration and repair of damaged tissues. Hydrogel helps to maintain cell viability [54]. Cell viability depends on several important properties of hydrogels, including cell source, pore size, structure, growth factor loading, oxygen, and nutrient availability [55].

### 3.4. Degradation

The hydrogel structure contains labile bonds that lead to their destruction in an aqueous environment or under the action of enzymes and are regulated by various external and internal factors, ultimately causing their degradation. The degree and rate of biodegradation of hydrogels are of critical importance in tissue engineering. Since hydrogels serve as a medium for tissue growth, they must eventually undergo degradation [56]. The degradation depends on several factors such as hydrophilicity, molecular weight, and the interaction of the polymer with water. Other environmental factors such as pH and temperature can also control degradation through simple solubilization. Moreover, hydrogels can also be degraded via enzymatic hydrolysis, which occurs through a group of hydrolases that catalyze the hydrolysis of C-C, C=O, and C-N bonds [57].

### 3.5. Swelling

Swelling of hydrogels occurs in three stages: (1) diffusion of water into the hydrogel matrix, (2) relaxation of the polymer chains followed by (3) expansion of the hydrogel network [58]. Swelling is measured by the swelling ratio, which is defined as the ratio of the swollen gel mass to the dry mass. Swelling kinetics and equilibrium are influenced by various factors such as cross-linking ratio, chemical nature of polymers, ionic environment, and temperature. Cross-linking affects the swelling ratio of a hydrogel as highly cross-linked structures have a lower swelling ratio and vice versa. Chemical structure also has a significant function on swelling property due to the hydrophilic and hydrophobic groups present in the polymer chains. Hydrogels containing more hydrophilic groups swell more compared to hydrogels containing more hydrophobic groups. pH-sensitive hydrogels swell due to ionization of hydrophilic groups with a change in pH level. Ionization creates electrostatic repulsion between like charges on the polymer and breaks secondary bonds between polymer chains [57,59,60].

Hydrogels can absorb and release drugs, creating a moist environment that stimulates cellular activity and tissue regeneration in cases of edema and fluid retention. Hydrogels can swell and contract without causing structural damage. Some hydrogels can swell irreversibly, changing their structure and characteristics due to physicochemical changes in the polymer network [61].

### 3.6. Wettability

Wettability is the ability of a liquid to spread completely over a solid, flat horizontal surface. Wettability determines the hydrophilicity and hydrophobicity of a biomaterial. This surface phenomenon depends on the chemical composition, surface roughness, and functional groups of the hydrogel [62]. Hydrophilic hydrogels absorb water, making them excellent for wound dressings, drug delivery systems, and tissue engineering applications [63].

## 4. Collagen-Based Hydrogels Cross-Linking Methods

### 4.1. Chemical Cross-Linking

**Figure 5:**
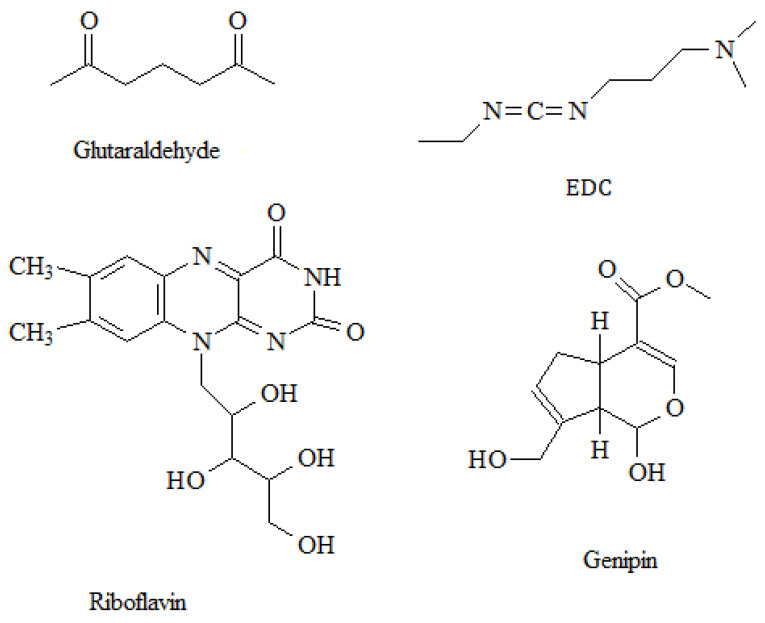
Most used collagen cross-linkers.

One of the collagen disadvantages is its biodegradability under the influence of proteolytic enzymes—collagenases. To slow down the biodegradation of collagen-based scaffolds, as well as to give them mechanical strength, cross-linking of collagen fibers with various chemical agents is used (Figure 5). Cross-linking agents interact with collagen through covalent, hydrogen or coordination bonds, and ionic interactions with functional groups of collagens, such as hydroxyl, amino, amide, and carboxyl, thereby changing the mechanical properties of collagen and also reducing the rate of thermal and enzymatic hydrolysis, which makes it possible to regulate biodegradation rate and obtain stable collagen-based biomaterials. In this way, three-dimensional cross-linked scaffolds of various architectures are obtained.

#### 4.1.1. Glutaraldehyde

**Figure 6:**
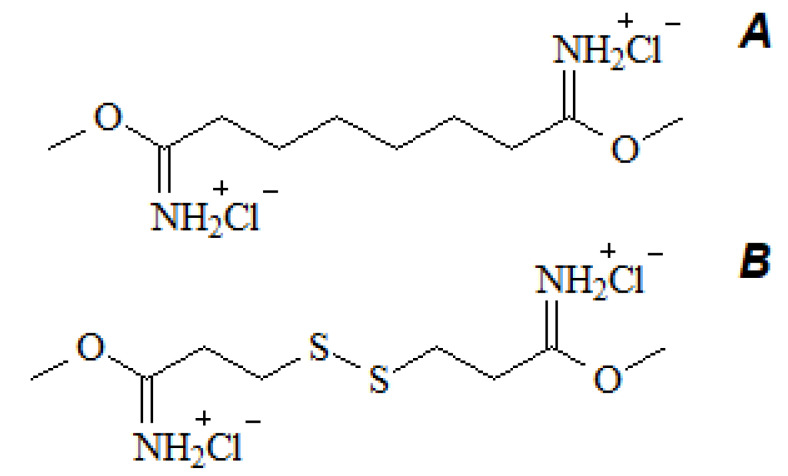
Structural formulas of (**A**)—DMS; (**B**)—DTBP.

Among the most used cross-linking reagents is glutaraldehyde, which is the “gold standard” in the development of tissue engineering constructions [64,65,66]. Because the molecule has two carbonyl groups that react with primary amino groups, it can function as a bifunctional cross-linker. However, toxicity and long-term calcification are the main disadvantages of glutaraldehyde cross-linked collagen [67,68,69,70]. In the case of glutaraldehyde cross-linked collagen, RGD sequences are shielded from fibroblast integrins, leading to decreased adhesion, migration, proliferation, and new collagen synthesis. Dimethyl suberimidate (DMS) has been proposed as an alternative to glutaraldehyde (Figure 6A) [71,72]. Collagen cross-linked with DMS turned out to be less toxic than collagen cross-linked with glutaraldehyde, but the thermal stability of the hydrogel deteriorated. In the search for reagents like DMS, but which would significantly increase the thermal stability of collagen, dimethyl 3,3′-dithiobispropionimidate (DTBP) was discovered (Figure 6B) [73]. The biocompatibility of cross-linked collagen samples studied by subcutaneous implantation in rats shows that although glutaraldehyde- and DTBP-treated collagen does not degrade within 4 weeks, DTBP-cross-linked collagen has a higher level of biocompatibility than glutaraldehyde-treated matrices, as demonstrated in structural studies and histological analysis [73].

#### 4.1.2. Dialdehyde Polysaccharides

In addition to glutaraldehyde, dialdehydes of various polysaccharides obtained during periodate oxidation according to the Malaprade periodate oxidation reaction are widely used [74,75]. Unlike low molecular weight organic bifunctional compounds, dialdehyde polysaccharides provide a wide choice of cross-linking agents, while the molecular weight and number of aldehyde groups are controlled during the reaction [76,77,78]. The main dialdehyde polysaccharides utilized as cross-linkers are dialdehyde carboxymethylcellulose [79,80], dialdehyde alginate [81,82], dialdehyde chitosan [83,84], and dialdehyde starch [85].

It is worth noting that not only polysaccharide dialdehydes can participate in cross-linking, but also protein dialdehydes, for example, heparin dialdehyde [86].

#### 4.1.3. Riboflavin

One of the most important water-soluble vitamins, riboflavin is involved in many biochemical processes. Riboflavin is used in the photo-cross-linking of collagen as a non-toxic alternative to glutaraldehyde. The combination of riboflavin and UV radiation results in collagen cross-linking with improved mechanical and thermal properties. In addition, riboflavin cross-linking demonstrates higher cytocompatibility with human mesenchymal stem cells compared to glutaraldehyde cross-linking [87]. Cross-linking with riboflavin and UV radiation is also used as a method of collagen-based implant fixation, such as intracorneal implants, as well as in the treatment of diseases such as keratoconus [88,89]. This method of fixation is promising as it reduces complications associated with suture placement, such as opacities and surface irregularities [89]. Promising results of UV cross-linking of collagen with riboflavin in degenerative eye diseases have shown that it may be effective in other corneal diseases.

#### 4.1.4. Genipin

Another commonly used cross-linking agent is genipin, a chemical compound found in the fruit of Genipa americana, which is an aglycone of iridoid glycoside (geniposide) [90]. Genipin reacts with primary amines; therefore, it is a cross-linking agent for compounds containing primary amino groups in their structure, such as proteins, collagen, and chitosan [91]. Compared with glutaraldehyde and other synthetic cross-linking agents, genipin is safe and non-toxic [92]. Also, a distinctive characteristic of genipin is the color change and the presence of fluorescence after cross-linking [93,94].

#### 4.1.5. Transglutaminase

Transglutaminase is an enzyme that catalyzes the formation of covalent bonds between free amino groups and γ-carboxyamide groups of glutamines [95,96]. Since collagen contains amino acids such as glutamine and lysine, the reaction takes place between their residues on adjacent protein fibers, thereby providing covalent amide bonds [97]. The most studied cross-linking agents are microbial transglutaminase [98,99,100] and tissue transglutaminase [101,102,103]. Films formed from collagen cross-linked with transglutaminase have improved physicochemical properties. Tensile strength and Young’s modulus were significantly higher than control samples containing uncross-linked collagen [97,104]. The usage of different transglutaminase concentrations to cross-link human collagen produces products with different degradation times and biocompatibility. Both hydrogels with a high cross-link density, which makes them more resistant to the action of collagenases, and with a low degree of cross-linking, which are more suitable for adhesion, proliferation, and migration of cells, including human osteoblasts, can be obtained [105]. Collagen substrates treated with transglutaminase also showed a higher degree of resistance to cell-mediated degradation by endogenous proteases than native collagen [106].

#### 4.1.6. Carbodiimides

All the above cross-linking compounds are reagents of non-zero length, that is, homobifunctional or heterobifunctional cross-linkers, which are a connecting bridge between polymer groups. Zero-length cross-linkers, in turn, activate the groups present in the substrates so that they become capable of attachment, i.e., the molecules are conjugated by a direct covalent bond without the use of a spacer. Carbodiimides are utilized to crosslink natural polymers and, in particular, collagen. The most popular is 1-ethyl-3-(3-dimethylaminopropyl) carbodiimide chloride (EDC), which is highly soluble in water, unlike other related compounds [107,108]. When cross-linking with EDC, the resulting scaffold shows increased resistance to temperature and enzymatic hydrolysis compared to non-cross-linked samples [109,110]. However, this mechanism results in a lower cross-link density than the use of other non-zero length bifunctional cross-linkers. But at the same time, a decrease in toxicity was shown, which in the case of using other cross-linkers was associated with an inflammatory reaction and reduced biological activity [111].

#### 4.1.7. Tannic Acid

**Figure 7:**
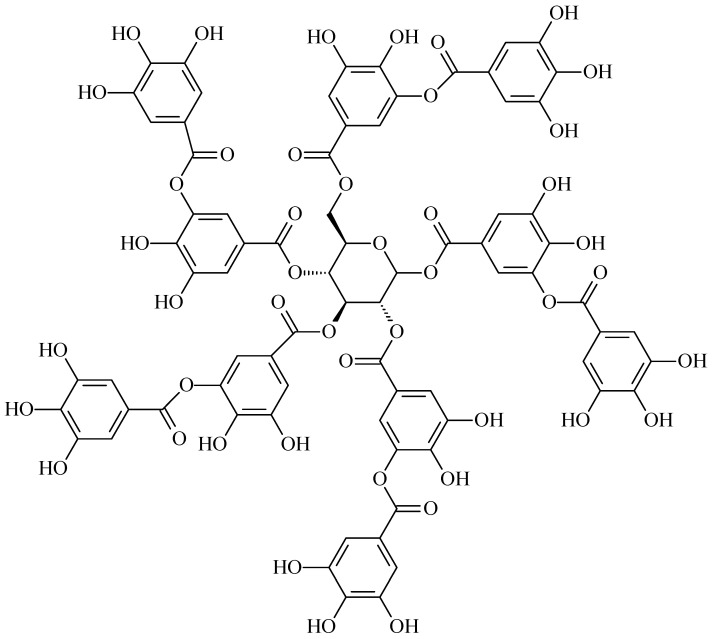
Structure of tannic acid.

Tannic acid is a type of plant polyphenol and has a variety of pharmacological properties, including anti-inflammatory, antioxidant, antitumor, neuroprotective, and antimicrobial activities (Figure 7) [112]. Tannic acid interacts with biomacromolecules such as chitosan, collagen, gelatin, and albumin through physical binding via hydrogen bonds, π-π stacking, and also through chemical reactions such as Michael addition, formation of Schiff bases, phenol-epoxy ring-opening reaction, etc. [113,114].

The binding of tannic acid and collagen is characterized by a high degree of affinity since the structural flexibility of collagen is compensated by the structural rigidity of phenolic compounds. Increasing the concentration of tannic acid causes a significant change in the conformation of the triple helix. Tannic acid binds tightly to collagen-like peptides through the formation of hydrogen bonds with side amino acid residues of arginine, as well as with the main chain at the amino acid residue of aspartic acid, and through hydrophobic interactions. The results show that the structural, thermal, and enzymatic stability of type I collagen cross-linked with tannic acid increases compared to native collagen. Collagen cross-linked with tannic acid exhibits high toxicity to cancer cells with minimal toxic effects on healthy cells [115,116]. Also, cross-linking with tannic acid inhibits the enzymatic degradation of collagen in micromolar concentrations by blocking collagenase access to the active sites of collagen chains due to the formation of hydrogen bonds and hydrophobic interactions [117]. The cross-linking of collagen with tannic acid is also affected by the presence of metal ions: Fe^3+^ and Ag^+^ increase the degree of cross-linking, while Zn^2+^ has an inhibitory effect. Ions such as Ce^3+^, Ca^2+^, and Na^+^ did not affect the degree of cross-linking [118]. In vivo studies in rats showed that a scaffold based on type I collagen cross-linked with tannic acid has good biocompatibility and is embedded into native tissue without fibrous encapsulation [119].

In addition to the cross-linking agents discussed above, other specific reagents listed in Table 2 can also be used. It is worth noting that most cross-linkers are bi- or polyfunctional and often contain phenolic residues in their structure.

Post-cross-linking of collagen hydrogels to improve their mechanical properties and cell proliferation is also of interest. Polyrotaxane is used as a post-cross-linking agent. Polyrotaxane is a type of mechanically linked molecule consisting of rings in which several rings are strung along a molecular axis and are prevented from separating by two bulky end groups [131]. Polyrotaxane, consisting of carboxymethylated α-cyclodextrins with a polyethylene glycol molecular axis, was used for cross-linking through reaction with amino groups in collagen. Post-cross-linking of collagen hydrogels improved the swelling ratio and mechanical properties such as viscoelasticity and tensile strength. Additionally, cell adhesion and proliferation were significantly improved on the surface of post-cross-linked collagen hydrogels compared to traditional cross-linking methods [132].

### 4.2. Physical Methods of Cross-Linking

#### 4.2.1. Radiation Cross-Linking

Physically cross-linked hydrogels are typically created through intermolecular, reversible interactions. A notable advantage of physical cross-linking is biomedical safety due to the absence of chemical cross-linkers, avoiding the potential cytotoxicity of unreacted cross-linkers.

One of the cross-linking methods is the use of ionizing radiation. Using this method, collagen with increased resistance to degradation and biocompatibility can be obtained, but the resulting collagen scaffold is characterized by a decrease in elastic properties, unsuitable surface architecture, and lack of proliferation of cells cultured on the scaffold. To avoid the above disadvantages, various ionization techniques or the use of other natural and synthetic polymers in addition to collagen can be used. For example, the incorporation of dextran into collagen and cross-linking by γ-irradiation produces a construction that exhibits a more porous structure with increased hydrophilicity, while at the same time, a decrease in cross-link density is found, resulting in larger pore sizes, high hygroscopicity, and reduced stiffness compared to the construction made from pure collagen. Accelerated degradation is observed when dextran is used, both in vitro and in vivo, leading to earlier integration with cells and tissues [133].

The ET-RaM technique for creating collagen hydrogels involves radiation-induced chemical cross-linking [134]. ^60^Co γ-rays, which are usually used to sterilize medical products, were used as a source of ionizing radiation. Although collagens are mainly destroyed by ionizing radiation [135,136,137], selected concentrations in water make it possible to create hydrogels based on both native and hydrolyzed collagen with high yield and high reproducibility. Collagens obtained by the ET-RaM method have the same properties as the native extracellular matrix. At the same time, the microtopography of the hydrogels themselves influenced the migration, proliferation, and differentiation of cells, which allows them to be used for inducing and controlling cell behavior [134].

It has also been shown that at low doses of gamma radiation (2–8 kGy), the structure of collagen remains unchanged, and its structural stability and compactness increase with increasing dose of gamma radiation [82].

#### 4.2.2. Ultraviolet Cross-Linking

The advantage of using ultraviolet (UV) irradiation to cross-link collagen is the absence of toxic cross-linking agents and the ease of control of the reaction. However, this method also has disadvantages: the probability of collagen fragmentation because of UV irradiation, which will affect the biocompatibility and degradation of collagen fibers [138]. Also, a partially denatured scaffold’s structure can lead to deterioration of stability in vivo and shrink when swelling [139]. Using denatured collagen scaffolds in vitro, cell adhesion and proliferation have been reported to be higher than with native collagen scaffolds.

Ultraviolet irradiation is also used in combination with cross-linking agents such as EDC [140] and riboflavin [89,141]. UV irradiation can be used as an adjunct to the cross-linking process with low doses of EDC, thereby not compromising the biocompatibility properties that occur at higher concentrations of EDC [141].

#### 4.2.3. Dihydrothermal Cross-Linking

This method aims to form cross-links in collagen when they are heated under vacuum. In this case, water molecules are removed, and polyester, polyether, and polyamide bonds are formed. Physical cross-linking using dihydrothermal treatment is utilized to stabilize fibrous collagen sponges. Dihydrothermally cross-linked collagen exhibits superior integration into surrounding tissue with minimal immune reactions and improved mechanical properties [142,143].

## 5. Modification of Collagen Hydrogels with Bioactive Molecules

**Figure 8:**
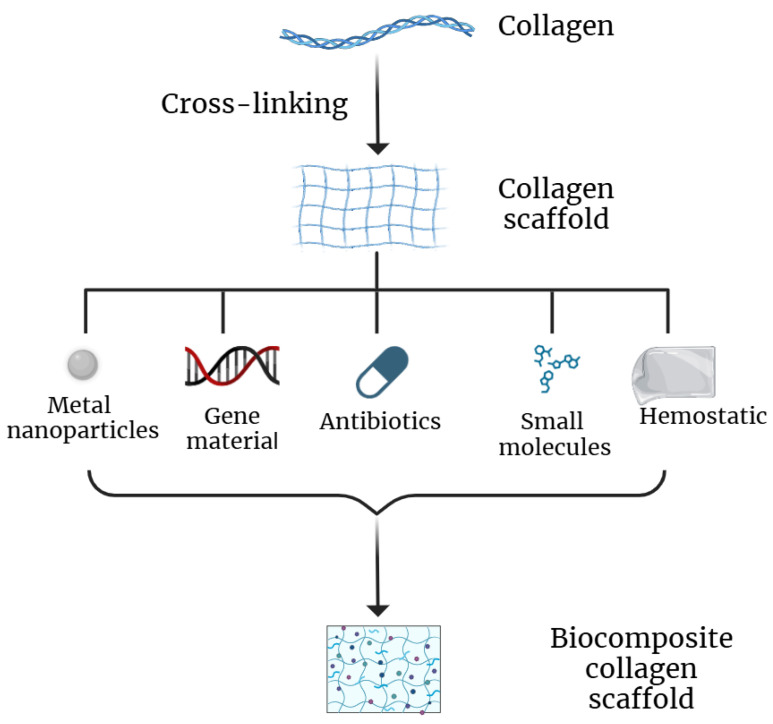
Different types of bioactive molecules that could be incorporated into collagen scaffolds.

Although collagen scaffolds are of interest as hydrogels for soft tissue replacement, as they have properties similar to the extracellular matrix, various additional drugs in the scaffold are required for effective regeneration (Figure 8). Antibiotics and their analogues to combat local bacterial infection, hemostatic drugs to stop bleeding, and various biologically active molecules, including proteins, DNA, etc. are necessary for improved tissue regeneration. This chapter discusses various drugs for incorporation in collagen scaffolds.

### 5.1. Antibiotics

Periprosthetic infection is an infection in the surgical site that develops after implantation of an endoprosthesis. Therefore, the incorporation of antimicrobial drugs into the structure of scaffolds is an important task for the prevention of secondary infection. The most widely used antibiotics are the aminoglycoside series with a wide spectrum of action—gentamicin, amikacin, etc. The incorporation of gentamicin in the collagen matrix does not affect the mechanical properties and cytotoxicity of the scaffold in vitro, and the release of the antibiotic is not limited in any way [144]. The second most applicable are cephalosporin antibiotics. For example, collagen cross-linked with hexamethylene diisocyanate with the incorporation of cefaclor, a second-generation cephalosporin antibiotic, was effective against *Escherichia coli* and *Staphylococcus epidermis* [125].

Antibiotics from the group of tricyclic glycopeptides are also promising for use in scaffolds. In the work by Thapa et al. [145], liposomes with the glycopeptide antibiotic vancomycin incorporated into a collagen scaffold were used. This was shown to be effective against methicillin-resistant *Staphylococcus aureus*. The encapsulation of vancomycin in liposomes allows for a long period of time to synergistically enhance the antibacterial effects of the composition both in vitro and in vivo. Moreover, increased antibacterial efficacy was evident even after repeated bacterial inoculations as a drug for the effective treatment of persistent wound infections.

Mupirocin is a broad-spectrum antibacterial and bactericidal agent for external use. Mupirocin, loaded into silica microparticles by the sol-gel method and uniformly distributed in a collagen scaffold, showed good antibacterial properties in in vivo studies, as well as excellent water absorption capacity, adhesion, and reduction in wound healing time [146]. Mupirocin incorporated in chitosan microspheres, in combination with *Piper betle* extract and enclosed in a collagen scaffold, showed satisfactory combination therapy for wound healing. Low molecular weight chitosan was chosen for its antibacterial activity against both Gram-positive and Gram-negative bacteria. In vivo studies indicate a potential effect of mupirocin, *Piper betle* extract, and chitosan on wound healing efficiency, which was observed in 90% of experimental rats on day 15 of the study. Histopathological examination further revealed collagen deposition, fibroblast proliferation, and the absence of inflammation, indicating effective wound healing after implantation of the scaffold with the drug combination [147].

### 5.2. Nanoparticles

Metal nanoparticles. Today, the problem of antibiotic bacterial resistance is extremely relevant for healthcare. In this regard, alternative strategies for treating bacterial infections without the use of antibiotics are being developed. Nanoparticles of various metals, which demonstrate increased antibacterial activity, are of great interest today [148]. Silver nanoparticles are among the most popular metal nanoparticles with antibacterial properties. As is known, additives of silver nanoparticles to antimicrobial drugs increase sensitivity by an average of 20% [149]. The antibacterial effect of silver nanoparticles is mainly due to the slow release of free silver ions from the nanoparticles. Silver ions have a positive charge and are absorbed by the negatively charged surface of the bacterial membrane.

Collagen-based scaffolds with silver nanoparticles stabilized by starch showed increased antibacterial effectiveness against both Gram-positive and Gram-negative bacterial strains compared to a scaffold not containing nanoparticles. Although the tensile strength of the scaffolds decreased with the addition of starch-coated silver nanoparticles, the value of Young’s modulus increased, indicating increased elasticity. Such scaffolds can be used to replace muscle tissue and heart valves [150]. Silver nanoparticles stabilized by polyethylene glycol and Triton X-100 and incorporated into a collagen scaffold also showed antibacterial effectiveness against both Gram-positive and Gram-negative bacteria. At the same time, the combination of surfactants increases the elasticity of the construction, making it possible to carry out surgical manipulations during implantation [151]. Collagen scaffolds with silver nanoparticles were obtained by 3D printing, followed by UV irradiation. This method allows for controlling the silver nanoparticle size, depending on the UV radiation interval. A larger amount of silver nanoparticles leads to more thermal stability of collagen, as well as resistance to the action of collagenase [152]. Collagen hydrogel loaded with silver nanoparticles and *Cannabis sativa* oil was developed without the use of chemical cross-linking agents and possessed a persistent antimicrobial effect against Gram-positive (*S. aureus*) and Gram-negative (*P. aeruginosa*) bacteria. At the same time, the hydrogel demonstrated improved mechanical properties and resistance to enzymatic hydrolysis by collagenase. The use of *Cannabis sativa* oil in the hydrogel showed improved antioxidant properties and biocompatibility [153]. Moreover, all scaffolds showed bactericidal activity against Gram-positive and Gram-negative bacteria.

In addition to the widely utilized silver nanoparticles, other less common metals and their oxides can be used for wound healing and antibacterial effects. An analogue of silver-based particles can be selenium nanoparticles, an element with great antimicrobial potential. In the work of Stevanović et al., selenium nanoparticles were developed and loaded into a collagen matrix. The resulting scaffold demonstrated significant antibacterial activity against Gram-positive strains, as well as against *Candida albicans* [154].

A study of molybdenum oxide nanoparticles incorporated into a collagen scaffold showed that they almost doubled cell adhesion and migration compared to pure collagen and pure molybdenum oxide in vitro. The composite material also promoted neovascularization and re-epithelialization. In vivo results on model animals showed that complete healing of wounds occurred on average on the 15th day [155].

Magnetic nanoparticles. Magnetic nanoparticles are used in hydrogels to replace VML for alignment of the structure, especially for injection routes. Collagen-based hydrogels obtained by gelation in the presence of cations, with incorporated magnetite nanoparticles, demonstrated properties similar to those of muscle tissue, as well as high biocompatibility, both in vitro and in vivo. Muscle cells were able to colonize the hydrogel, which led to a promising result for regenerative purposes [156]. Superparamagnetic iron oxide nanoparticles (SPIONs) incorporated into collagen scaffolds demonstrated retention of magnetic characteristics without changing cell viability, adhesion, and proliferation compared to free collagen. The incorporation of SPIONs allows for the production of scaffolds by electrospinning [157]. The presence of magnetic nanoparticles also allows for the controlled release of drugs. Paramagnetic iron oxide nanoparticles incorporated into collagen-based gel. In [158], fluorescein was used as a model drug. Fluorescein release was clearly triggered and controlled by consecutive magnet applications. The presence of iron nanoparticles did not affect cell viability. Iron nanoparticles can also be used for contrast imaging in magnetic resonance imaging (MRI). Collagen-based scaffolds loaded with iron oxide nanoparticles were implanted in rats. The result showed that the incorporation of iron oxide particles improved the contrast of implants by MRI, providing information on their location, shape, and degradation [159].

Polymeric nanoparticles. Polymer nanoparticles can be used for prolonged release of drugs from scaffolds. They have advantages such as biocompatibility, biodegradability, and lack of toxicity. The incorporation of active substances in polymer nanoparticles also allows for increased drug solubility, changes in its pharmacokinetics, and reduced side toxicity. The most studied polymer carrier is poly(lactide-co-glycolide) (PLGA). It can be used not only as carriers of drugs, but also of genetically engineered structures (RNA, proteins, etc.). Moreover, some drugs based on PLGA are approved by the FDA for medical use.

The use of PLGA nanoparticles with entrapped fish oil, loaded into collagen-based scaffolds, showed an increase in hydroxyproline production, as well as the expression of the CCR5 (adhesion protein), EGF (epidermal growth factor), TGFB1 (transforming growth factor, beta-1), and CCL5 (chemokine (C-C motif) ligand 5) genes compared to the control in a porcine skin wound healing model. PLGA nanoparticles with the commercially available antibacterial and bactericidal agent with a broad spectrum of action mupirocin were used as a control [160].

PLGA nanoparticles loaded with human antimicrobial peptide beta-defensin-2, impregnated into a collagen/chitosan scaffold, were developed in the work [161]. The scaffold had low matrix degradation, optimal porosity, and sustained peptide release. In vitro studies showed that the scaffold was biocompatible, accelerated cell migration, and promoted angiogenesis. The scaffold also showed significant antimicrobial activity against *S. aureus*, *E. coli*, and *P. aeruginosa*. In vivo studies showed accelerated healing in a rat diabetic wound model.

### 5.3. Biologically Active Molecules

To combat bacterial infection, drugs other than antibiotics and metal nanoparticles can be used in the scaffold. For example, silver sulfadiazine is active against *Escherichia coli*, *Proteus* spp., *Staphylococcus* spp., and *Klebsiella* spp. The bactericidal properties are due to the activity of silver ions, which are released in the wound bed because of the sulfadiazine silver salt dissociation. Alginate microspheres loaded with silver sulfadiazine demonstrated sustained in vitro drug release from the scaffold and activity against *Klebsiella pneumoniae*, *Escherichia coli*, *Pseudomonas aeruginosa*, and *Staphylococcus aureus* [162].

Quaternary ammonium bases also have antibacterial activity against Gram-positive bacteria. For example, quaternary ammonium silane is active against *Enterococcus faecalis* and *Candida albicans* [163]. The incorporation of quaternary ammonium silane in a collagen scaffold cross-linked with riboflavin in the presence of d-alpha-tocopheryl poly(ethylene glycol)-1000-succinate does not change the tertiary structure of collagen, while the scaffolds have antibacterial activity [164].

Polyhexamethylene biguanidine (PHMBG) may be a promising compound as an antiseptic and fungicide. PHMBG incorporated in the collagen scaffold is released within 5 days and destroys pathogenic microbiota in the wound bed. Cytocompatibility and efficacy against Gram-positive and Gram-negative bacteria demonstrated that the PHMBG-containing collagen scaffold may be a promising alternative for the treatment of infected wounds [165].

Another promising cross-linking agent and physiologically active substance is curcumin, which has potential anti-inflammatory, antitumor, antibacterial, and other properties. In work by Dharunya et al. [127], curcumin was used as a cross-linking agent and drug. The results confirmed that cross-linking with curcumin did not cause any structural changes in collagen. Curcumin cross-linked collagen aerogels demonstrated high antiproteolytic and antimicrobial activity. Curcumin loaded into lipid nanoparticles did not affect such properties of cryostructured collagen as porosity, swelling coefficient, degradation ability, and cytotoxicity. Due to encapsulation, curcumin was released from the scaffold rather slowly, which can potentially provide a long-term therapeutic effect [166]. Combinations of various drugs can also be used in the scaffold. For example, the simultaneous utilization of tannic acid and chlorhexidine bigluconate in matrices based on type I atelocollagen (a low-immunogenic collagen derivative) as active substances with a wide spectrum of antimicrobial activity, as well as cross-linking agents [167].

Collagen-based scaffolds can also serve as a platform for the delivery of proteins and genetic material. In work by Raftery et al. [168], chitosan nanoparticles with plasmid DNA were used as part of a collagen scaffold. The transfection efficiency is more than 45%, which is comparable to the “gold standard” transfection—polyethylenimine. Chitosan nanoparticles with plasmid DNA were incorporated into collagen-based scaffolds, and stable transgene expression from mesenchymal stem cells cultured on the scaffold was maintained for up to 28 days. These results demonstrate that the system has potential for multiple therapeutic applications.

Cu^2+^ chelated epigallocatechin gallate (EAC NPs) nanoparticles are promising agents for radical scavenging, anti-inflammatory, antibacterial, and vascularization promotion. EAC NPs exhibit a strong antibacterial effect against *E. coli* and *Staphylococcus aureus*, and protective effects against H_2_O_2_-induced oxidative stress in vitro. Collagen loaded with EAC NPs maintains a porous structure similar to free collagen scaffold. In vivo studies show that collagen scaffold with EAC NPs can promote wound healing in a rat wound model. The composite collagen scaffold loaded with EAC NPs has multiple life-saving functions and is a promising wound care dressing [169].

### 5.4. Hemostatic Drugs

Uncontrolled bleeding is the leading cause of death in patients with severe trauma and during surgery. Therefore, when replacing and restoring volumetric muscle losses, especially during surgery and sometimes after, the use of hemostatic drugs is required. Collagen scaffolds themselves are good hemostatic agents and are sold under various brand names: «Alvanes» (VladMiva TM, Belgorod, Russia), «SpongeCol» (Advanced BioMatrix, Carlsbad, CA, USA), «Collaclot» (Stratton Technologies, Singapore), etc. Most collagen-based hemostatic sponges have a good hemostatic effect due to the presence of pores, hygroscopicity, and the ability to maintain hemostasis [170]. However, surface heterogeneity and toxicity of cross-linking agents can lead to negative consequences. Therefore, today both new methods for the synthesis of hydrogels and hemostatic additives to already developed hydrogels are being studied.

In work by Shi et al. [171], a scaffold based on collagen, liposomes with incorporated tissue factor (TF), and silica nanoparticles was used. This combination of active substances demonstrated increased procoagulant efficacy in the absence of cytotoxicity. These results suggest that collagen hydrogels with embedded TF liposomes and silica nanoparticles can serve as a platform for the development of an effective collagen-based hemostatic composite.

The utilization of a combination of collagen hydrogel with tranexamic acid, a fibrinolysis inhibitor, looks promising. In in vivo experiments on the Wistar rat line, in models of standard liver injury and standard spleen injury, a collagen hydrogel with 9% tranexamic acid and commercially available collagen sponges from Belkozin (Russia) and Zelenaya Dubrava (Russia), as a control, were studied. The most effective, in terms of the difference in the rate of blood flow from the wound and the rate of its absorption, is a hemostatic sponge with 9% tranexamic acid [172].

In work by Jiang et al. [173], a new hemostatic sponge was developed based on human collagen cross-linked with glutamine transaminase, the surface morphology of which was optimized using two-stage freezing. The resulting sponge showed good biocompatibility in cytotoxicity tests and implantation and had a significant hemostatic effect in models of auricular artery and liver injury.

The synthesis of a hemostatic sponge based on recombinant collagen is interesting. In work by He et al. [45], a new recombinant collagen was expressed by Pichia pastoris with an optimized amino acid sequence design. It had better hydrophilicity and adhesion to blood cells, which could potentially further improve the hemostatic effect of the recombinant collagen sponge. Collagen hemostatic sponges were prepared by the carbodiimide method by cross-linking EDC in combination with N-hydroxysuccinimide. The resulting sponge showed high platelet adhesion and a rapid coagulation effect in vitro. The hemostasis effect in the animal model was also significantly more effective than other sponges on the market.

## 6. Limitations and Challenges of Collagen-Based Scaffolds for Volumetric Muscle Loss Regeneration

This review provides a comprehensive summary of collagen-based hydrogel development to replace volumetric muscle loss, including collagen cross-linking strategies, rheological properties of scaffolds, drug incorporation, and tissue engineering applications. Although many breakthroughs have been made in the development of these hydrogels, the lack of multimodal drug release remains a drawback. The optimal method is to release drugs at the desired stage of tissue repair at a certain rate. In the first stage, antibiotics and anti-inflammatory drugs are locally released, and in the second stage, drugs that help in the regeneration of new tissues are released. Also, despite the growing interest in collagen scaffolds, precise control of their architecture, such as pore size, wall thickness, surface roughness, etc., is required. Despite a number of techniques developed to control scaffold architecture, such as laser patterning, electrospinning, 3D printing, and cryostructuring, predicting and designing the macrostructure remains a challenge. Therefore, a key challenge in the field of collagen hydrogel design is determining the relationship between the properties of hydrogel components and their biological effects, as well as establishing design strategies for the optimization and standardization of collagen scaffolds.

## 7. Conclusions and Future Prospects

Collagen scaffolds for soft tissue replacement are promising developments, and their market is growing year by year. The global market size is expected to reach USD 6.52 billion by 2032. The need for scaffolds is driven by the increasing incidence of chronic diseases and injuries, including osteoarthritis, volumetric muscle loss, and fractures. Collagen scaffolds have several advantages: ease of synthesis, high water content, safety in use, control of the biodegradation rate due to the degree of cross-linking, etc. The choice of cross-linking agent allows control of parameters such as elasticity, mechanical strength, biodegradation rate, and toxicity. In addition, scaffolds can be modified for the controlled release of drugs, growth factors, genetic material, and other biologically active substances. The incorporation of such substances is determined by the stages of tissue regeneration. At the first stage, it is necessary to administer hemostatic and antibacterial drugs; at the second, growth factors and genetic material for the regeneration of own tissue. Biocomposite scaffolds, containing necessary substances, which are released in a controlled manner at each stage, become universal depots and do not require additional medications.

## Figures and Tables

**Table 1:** Some commercially available collagen-based products.

Product	Manufacturer	Composition	Application
INFUSE^®^ BoneGraft	Medtronic (Dublin, Ireland)	Collagen sponge with recombinant bone morphogenetic protein 2 (rhBMP-2)	Sinus-lifting, restoration of alveolar ridge defects
InductOS^®^	Medtronic (Dublin, Ireland)	Dibotermin alpha on collagen sponge	Healing of tibial fractures
Helistat^®^	Integra LifeSciences Corporation (Princeton, NJ, USA)	Bovine collagen	Hemostatic sponge
NeuraGen^®^	Integra LifeSciences Corporation (Princeton, NJ, USA)	Collagen and chondroitin-6-sulfate	Implant for repairing peripheral nerve ruptures
GRAFTJACKET™	Wright Medical Group N.V. (Portage, MI, USA)	Human dermal collagen	Provides additional support, protection, and strengthening of tendons and ligaments
Resolve Matrix™	Parametrics Medical (Leander, TX, USA)	Porcine collagen	Treatment of local wounds
«Stimul-OSS»	Belkozin (Luga, Russia)	Collagen, hydroxyapatite, chlorhexidine	Various types of surgical interventions in dentistry and maxillofacial surgery
«Bioplast-Dent»	VladMiVa (Belgorod, Russia)	Type I–III collagens	Replacement of bone tissue defects
«Alvanes»	VladMiVa (Belgorod, Russia)	Lyophilized collagen with lincomycin and lidocaine	Treatment and prevention of inflammatory complications in surgical dentistry and periodontology
BIOSTEP Ag Collagen Matrix	Smith & Nephew (London, UK)	Collagen with silver	Treatment of acute and chronic full and partial-thickness wounds
Collprotect^®^	BOTISS Biomaterials (Zossen, Germany)	Native porcine collagen	Regeneration of dental bone and soft tissues
Mucograft^®^	Geistlich Pharma AG (Wolhusen, Switzerland)	Highly purified porcine collagens types I and III without cross-links	Regeneration of soft tissues in the maxillofacial area

**Table 2:** Collagen cross-linking agents.

Cross-Linking Agent	Structure	Ref.
Squaric acid	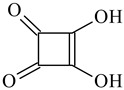	[120]
1,4-Butanediol diglycidyl ether	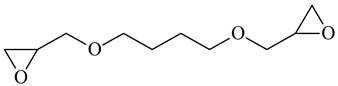	[121]
2-Iminothiolane (Traut’s Reagent)	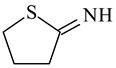	[122]
Diphenylphosphoryl azide	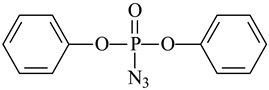	[123]
Nordihydroguaiaretic acid	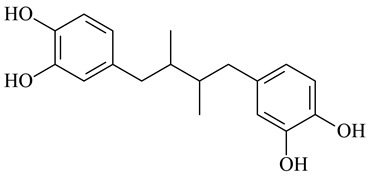	[124]
Hexamethylene diisocyanate	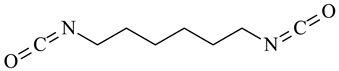	[125]
Epigallocatechin gallate	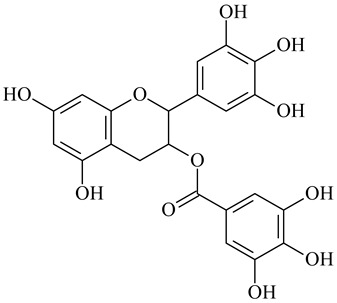	[126]
Curcumin	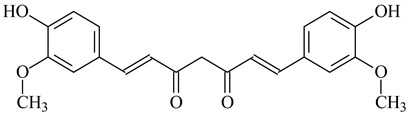	[127]
Gold nanoparticles with tiopronin	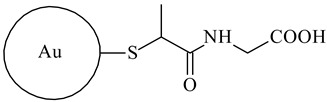	[128]
Ferulic acid	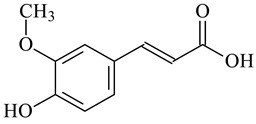	[129,130]
Caffeic acid	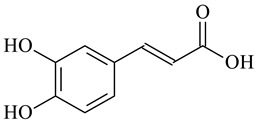	[124]
Gallic acid	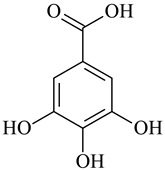	[129,130]
Ellagic acid	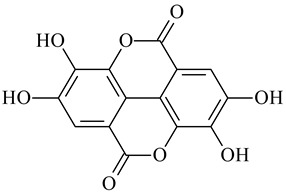	[130]

## Data Availability

Data sharing not applicable. No new data were created in this study. Data sharing is not applicable to this article.
